# Cocirculation of 2 Genotypes of Toscana Virus, Southeastern France

**DOI:** 10.3201/eid1303.061086

**Published:** 2007-03

**Authors:** Remi N. Charrel, Arezki Izri, Sarah Temmam, Pascal Delaunay, Isabelle Toga, Henri Dumon, Pierre Marty, Xavier de Lamballerie, Philippe Parola

**Affiliations:** * Université de la Méditerranée, Marseille, France; † Assistance Publique – Hopitaux de Marselle, Timone, Marseille, France; ‡ Unité de Formation et de Recherche, Bobigny, Paris, France; § Centre Hospitalier Universitaire l’Archet, Nice, France; Assistance Publique – Hopitaux de Marselle, Nord, Marseille, France; 1 Equally participated in this work.; 2 Equally participated in the design and supervision of this study.

**Keywords:** sandfly, phlebovirus, meningitis, France, dispatch

## Abstract

Toscana virus (TOSV), an arthropodborne phlebovirus transmitted by sandflies, can cause febrile illness and meningitis. The vector of TOSV in France was unknown. We detected TOSV RNA in 2 (female *Phlebotomus perniciosus*) of 61 pools of sandflies captured in southeastern France. Two genotypes of TOSV were identified.

Toscana virus (TOSV) is an arthropodborne virus first isolated in 1971 in central Italy from the sandfly *Phlebotomus perniciosus*, then from *Ph. perfiliewi* (*1*,*2*). In Spain, TOSV has been isolated from phlebotomine flies, but species identification was not performed (*3*). TOSV has not been detected in or isolated from sandflies in France.

Most clinical and epidemiologic studies on TOSV have been conducted in Italy, although data from other Mediterranean countries have recently been published (*4*). TOSV has a tropism for the central nervous system and is a major cause of meningitis and encephalitis in the countries in which the virus circulates. Cases of TOSV infection acquired in the south of France indicate that TOSV circulates in the region (*5*,*6*). The aim of our study was to identify the presence of TOSV in *Phlebotomus* spp. collected in southeastern France and to investigate French TOSV at the genetic level. For that purpose, we compared viral sequences from sandflies and from French patients with other sequence data previously reported from Italy and Spain.

## The Study

**Figure 1:**
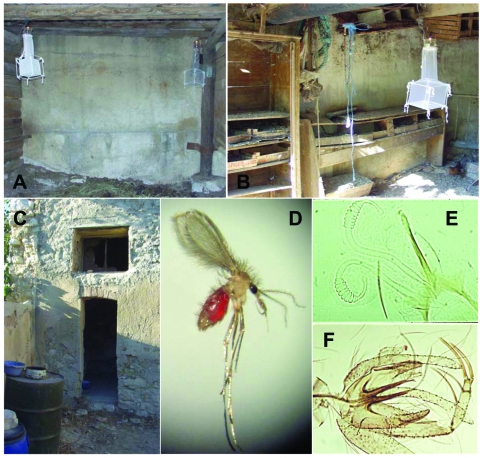
CDC light traps, adapted for sandfly trapping, placed in horse stables (A), near rabbit hutches and henhouses (B), and in quiet places in the shade of human habitations, where dogs sleep (C). Engorged female *Phlebotomus perniciosus* sandfly trapped in a horse stable (D); spermatheca of *Ph. perniciosus* female after dissection (E); genitalia of *Ph. perniciosus* male after dissection (F).

Sandflies were trapped during 4 days in July 2005 in 2 cities in southern France (Marseille and Nice) by using techniques previously reported (*7*) (Figure 1). Adult flies shelter during the day in dark, quiet, and humid places, and eggs are laid in terrestrial microhabitats rich in organic matter that provides food for larvae, including soils containing herbivorous animal feces. Therefore, traps were placed at dawn in or near animal housing facilities (horse stalls, rabbit hutches, hen houses) or in areas where visceral canine leishmaniasis is endemic (*8*). Each morning, sandflies were collected and identified morphologically, by dissecting genital organs, according to morphologic taxonomic keys (*9*). Sandflies were pooled (up to 30 individual flies) according to trapping origin, species, and gender (Table) in 1.5-mL tubes and processed as described (*7*).

**Table:** Capture of sandflies and identification

Trapping region	Species	Sex	No. sandflies	No. pools
Marseille	*Phlebotomus perniciosus*	M	291	17
		F	185	15*
		Unknown	7	3
	*Ph. ariasi*	M	2	2
		F	1	1
	*Ph mascitii*	M	1	1
		F	3	3
	*Phlebotomus* sp.	M	3	3
		F	2	1
		Unknown	4	4
	Total Marseille		499	50
Nice	*Ph. perniciosus*	M	78	5
		F	32	3
	*Phlebotomus* sp.	M	43	2
		F	23	1
	Total Nice		176	11
Total			675	61
*Two pools (AK and AR) out of these 15 pools were positive. AK sandflies were trapped in the Maillane District (43°17′51” N, 5°29′43” E). AR sandflies were trapped in the Mont d’Or District (43°22′52” N, 5°22′17” E**).**				

A variety of primers targeting different genes were used: primers specific for TOSV in the polymerase gene (*10*) and primers designed in this study from the alignment of nucleoprotein sequences of selected phleboviruses (retrieved from GenBank) specific for the species *Sandfly fever Naples virus* (SFNV-S1 [5′-CTTYTTRTCYTCYCTRGTGAAGAA-3′], SFNV-R1 [5′-ATGATGAAGAARATGTCAGAGAA-3′], SFNV-S2 [5′-GCRGCCATRTTKGGYTTTTCAAA-3′], SFNV-R2 [5′-CCTGGCAGRGACACYATCAC-3′]). The reverse-transcription–PCR (RT-PCR) was performed by using the Access RT-PCR kit (Promega, Madison, WI, USA) according to the manufacturer’s recommendations. L and S RNA primers were used at 0.8 μmol and 0.4 μmol per reaction, respectively. The cycling program of the RT-PCR reaction consisted of 48°C for 45 min and 94°C for 2 min, followed by 40 cycles at 94°C for 30 s, the annealing temperature for 1 min, and 68°C for 45 s, with a final elongation step at 68°C for 7 min.

Using ground material obtained from pools AK and AR and patients’ samples, we injected Vero-E6 cells (protocol available on request). Most sandflies belonged to the *Ph. perniciosus* species (Table, Figure 1), 483 (96.8%) in Marseille, 110 (62.5%) in Nice. Two other species of phlebotomine flies were identified, *Ph. mascitii* (n = 4, 0.8%), and *Ph. ariasi* (n = 3, 0.6%) in Marseille. In addition, 123 *Sergentomyia minuta* were identified and led to the detection of TOSV RNA, as recently reported (*7*). Finally, 61 pools of *Phlebotomus* spp. were organized to be tested for the presence of TOSV RNA.

We detected TOSV RNA by both PCRs in 2 pools (AK and AR) of female *Ph. perniciosus* trapped in the neighborhood of Marseille. AK and AR sequences were distinct from each other (1.4% and 1% nucleotide divergence for S and L RNA sequences, respectively), from other sequences available in GenBank, and from those determined during this study or in previous studies (0.7%–21.5% and 17.4% nucleotide divergence for S and L RNA sequences, respectively) (*7*).

**Figure 2:**
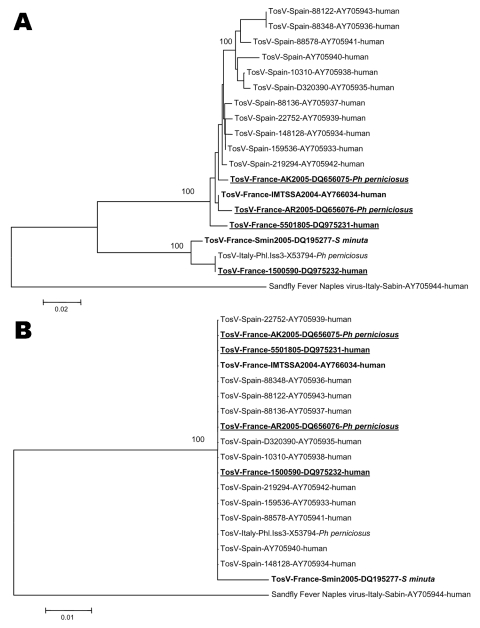
Phylogenetic trees based on nucleotide (A) and amino acid (B) sequences in the nucleoprotein gene of phleboviruses within the species Sandfly fever Naples virus. Sequence information corresponds to virus/country of origin/strain/GenBank accession no/host. Sequences representing French TOSV are in **boldface.** Sequences determined in this study are underlined. Sequence alignment was achieved with ClustalX 1.81 with sequences from other phleboviruses retrieved from GenBank. Accession numbers of GenBank sequences used for genetic analyses are indicated. Phylogenetic studies were conducted by using MEGA version 3.0 (*11*). Genetic distances were calculated with the pairwise distance and Jukes-Cantor methods at the nucleotide and amino acid level, respectively. Phylogenetic trees were constructed with the neighbor-joining method. The robustness of the nodes was tested by 500 bootstrap replications.

Sequences from 3 TOSV-infected patients who lived in the region of Marseille were included in the genetic analysis. For 2 patients, sequences were obtained after virus isolation in Vero cells: strain IMTSSA/2004 (GenBank accession no. AY766034) (*6*) and strain 1500590 (GenBank accession no DQ975232–975233, this study). Another sequence was determined from direct amplification of a cerebrospinal fluid sample (GenBank accession no. DQ975231, this study). Nucleotide distances (Appendix Figure) clearly allowed discrimination of the 2 genotypes previously described as the Italian and the Spanish genotypes by reference to the origin of viruses. Sequences obtained from sandfly and clinical material collected in France indicated that both genotypes are circulating in southern France. Indeed, *Ph. perniciosus* specimens AK and AR and human samples 5501805 and IMTSSA/2004 displayed high identity levels with TOSV of Spanish origin. In contrast, *S. minuta* specimen Smin2005 and human sample 1500590 were more closely related to the Italian strain Iss.Phl3 (Appendix Figure). Phylogenetic analysis of nucleotide sequences (Figure 2A) showed that Spanish, French, and Italian TOSV and SFNV formed a cluster that included members of the species *Sandfly fever Naples virus*, for which genetic data were available. Such analysis confirmed that the 2 French TOSV sequences detected in *Ph. perniciosus* (AK and AR) and the 2 TOSV sequences from clinical samples (IMTSSA2004 and 5501805) grouped with Spanish TOSV (100% bootstrap support), while *S. minuta* specimen Smin2005 and human sample 1500590 clustered with Italian TOSV sequences. Topology analysis indicated that the 4 French TOSV most closely related to Spanish TOSV root the Spanish sequences (Figure 2A). Together, all these sequences displayed a cline topology. Further studies, with more sequences, are needed to investigate this finding. As previously reported (*3*), despite significant variability between TOSV isolates at the nucleotide level (up to 13.2%), phylogenetic analysis of amino acid sequences did not allow discrimination between the Italian and Spanish genotypes (Appendix Figure, Figure 2B). Indeed, in the nucleoprotein region considered for analysis, all TOSV amino acid sequences were 100% identical, except that obtained from *S. minuta*, which diverged by 1 amino acid (data not shown).

## Conclusions

This study provided genetic evidence, from the analysis of 2 independent genes, that TOSV circulates in populations of *Ph. perniciosus* in southeastern France. The absence of detection of TOSV in the Nice region may be due to the small number of sandflies collected. A similar study aimed at collecting a larger number of sandflies is therefore necessary to address whether TOSV circulates in the region of Nice and whether, if it does, it belongs to the Spanish genotype, the Italian genotype, or another yet unrecognized genotype.

The rate of infection observed with TOSV in sandflies collected during this study (2/675) is of the same magnitude of that reported in Spain (*3*) and lower than rates reported in Italy (*2*). The distribution and respective frequency of sandfly species in France (this study) and Spain (*3*) were different: *Ph. papatasi* (6.8%) and *Ph. sergenti* (8.5%) were cataloged in Spain, not in France, whereas *Ph. mascitii* (5.9%) was identified in France, not in Spain. *Ph. perfiliewi*, known to replicate TOSV in nature in Italy, has not been identified in either French or Spanish studies.

In conclusion, we have provided the first evidence for the circulation of TOSV in *Ph. perniciosus* in the region of Marseille, southeastern France. We also determined that TOSV belonging to the 2 genotypes previously recognized in Italy and Spain circulates and causes human infections in southeastern France.

## Supplementary Material

Appendix Figure Genetic distances in the nucleoprotein gene between viruses within the Sandfly Fever Naples virus species. Diversity was calculated by the pairwise-distance algorithm implemented in the MEGA software program. (Reference: Kumar S, Tamura K, Nei M. MEGA3: integrated software for Molecular Evolutionary Genetics Analysis and sequence alignment. Brief Bioinform. 2004;5:150-63.)
