# Supplementary figures and images for: Cocirculation of 2 Genotypes of Toscana Virus, Southeastern France
Source: Emerg Infect Dis. 2007 Mar;13(3):465–8. doi: 10.3201/eid1303.061086 (PMC2725915; doi:10.3201/eid1303.061086)

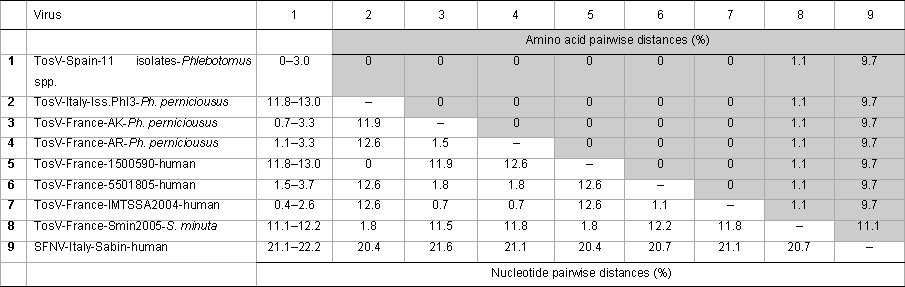

Supplement: Appendix Figure — Genetic distances in the nucleoprotein gene between viruses within the Sandfly Fever Naples virus species. Diversity was calculated by the pairwise-distance algorithm implemented in the MEGA software program. (Reference: Kumar S, Tamura K, Nei M. MEGA3: integrated software for Molecular Evolutionary Genetics Analysis and sequence alignment. Brief Bioinform. 2004;5:150-63.) [file 06-1086_app-s1.gif]
